# Mr. Kelson, on Vaccine Inoculation

**Published:** 1800-07

**Authors:** T. M. Kelson

**Affiliations:** Seven Oaks


### *To the Editors of the Medical and Phyfical Journal.*

GENTLEMEN,

FROM my not having been in the habit of reading your Phyfical Journals, I remained till this day, (when a friend put the whole of them into my hands) nearly ignorant of the variety of debates which have taken place upon the important fubject of Vaccine Inoculation. Dr. Jenner's two firft publications, a paper of Dr. Pearfon's, and an early one of Dr. Woodville's, comprehended the whole of my reading on the difeafe; but, I have been by no means an idle practitioner in the bufinefs, and I have not failed to endeavour to fatisfy myfelf completely in every doubtful point regarding it: and I find, in that refpect, I have fucceeded quite as well as my brother inoculators. If you can afford me a page or two in your next Pamphlet, I will mention a few particulars which, I hope, may not be unfatisfactory. Early in laft Spring, Dr. Pearfon (of whofe acutenefs and indefatigable perfeverance too much cannot be faid) fent me fome vaccine virus, with which I inoculated feveral perfons; in two inftances only it took effect; on on a man and a little girl: The former about the ninth day became ill, and fo continued for feveral days, when a few *pink, fiery-looking bliſters* appeared on his body, and then followed a very numerous eruption, exactly like fmall-pox of the diftinct kind; they took the ufual courfe of variolous puftules, and the man did well. From one of thofe *pink veſications*, I inoculated his wife, and fhe had the *true cow-pox*, with only *local puſtules*: the man's fon, who never had the fmall-pox, remained with his father the whole time, but did not catch the diforder. From the woman I inoculated three children, and they all had eruptions of the *variolous kind*, and were very ill during the eruptive fever. From thofe I inoculated a few others, fome of whom had eruptions, and fome had not. At this time, I was alfo ufing matter which originated with the little girl, who took it when the man did, and which I foon learnt to prefer, fhe having the difeafe in the mildeft form poffible; and from this, in no one inftance did eruptions occur, in upwards of a hundred patients, from two weeks old to eighty years, whom I inoculated laft Spring. During the Summer I wholly difcontinued it; in the Autumn, through the kindnefs of Dr. Pearfon, I again obtained fome virus, and have inoculated as many more, without having one eruptive cafe. The facts I can ftate to be clearly demonftrated by my extenfive inoculation, are thefe: That the difeafe, when unattended with eruptions fimilar to variolous, (which, by the bye, notwithftanding fuch high authority as Dr. Pearfon's, I believe to make no part of it) is a *thouſand times more trifling than ſmall-pox*, fcarcely having had a patient fufficiently ill to prevent amufement or labour:—That the *local inflammation* of the arm is not to be dreaded; I never had occafion for any other application than a bit of rag, when the puftule has been rubbed off; that rag has been finged:—That it is not an *infectious diſeaſe*; to determine which, I felected about forty people in our workhoufe, and inoculated half of them, fome in both arms, and fixed them to fleep with thofe who had not had it; but in no inftance was it communicated to the others. I broke the puftules, and frequently made them fmell the parts, but to no effect. After giving the difeafe to the remainder, I, beyond cavil, afcertained it to be a *perfect ſecurity againſt the ſmall-pox*, for I immediately inoculated the whole party with the moft virulent variolous matter I could procure; but nothing enfued, except local fuperficial inflammation for the firft fix or feven days. I then introduced a wretched family, juft recovered from very bad fmall-pox, their dirty clothes unchanged, and divided them in different beds among them, but to no purpofe. I then inoculated with cow-pox an infant, and as foon as as I was fatisfied it had taken, I put it, and kept it in the bed with its fifter, who had the moft dreadful confluent fmall-pox, but no inconvenience enfued. Moft of thefe workhoufe children I have this fpring inoculated again, both with variolous and vaccine matter, but nothing happens: this fhews the vaccine effect to be a *lafting* fecurity againft *itfelf*, as well as the *fmall-pox*. I have inoculated feveral people with it, who have had the fmall-pox, but it has only proved, they are *not fufceptible* of the difeafe. If a perfon be inoculated with vaccine lymph, and the operation be repeated a few days after, if the firft have taken, as that advances, the fecond, though taken alfo, difappears. Befides the cafes above noticed, moft of the others whom I have inoculated, have had variolous matter inferted afterwards, for the fatisfaction of themfelves or friends.

I muft now fay fomething refpecting the cafes where *puftules* appear. I believe they are to be accounted for this way:—Originally, fome how or other, the vaccine virus gets intermixed with variolous; in this ftate it is inferted into the arm together; they are both acting on the furrounding parts, *locally and diftinctly*: if, perchance, the vaccine fermentation goes on the fafteft, *that* gives the ftimulus to the conftitution, and the cow-pox is produced; if the variolous do, the fmall-pox is the confequence. Should they happen to keep tolerable pace with each other, I think both get into the habit, as in the cafe of my firft patient. Now, in drawing a thread through this mixed local puftule, if you fuppofe it to be merely mixed, and not, if I may ufe the expreffion, chemically combined, (which by no analogy we can allow) one part of that thread may have particles of variolous, the other of vaccine virus adhering to it; and, thus account for my firft little girl's cafe, and thofe who followed, &c. All the local puftules I ever faw in thefe eruptive cafes, contained a fluid of a different *confiftence and appearance* from the true vaccine lymph, approaching very nearly to pus. If the thing could have been procured from *greafy-heeled horfes*, I fhould have had the means of propagating the difeafe fome months fooner than I had, as I left no experiment untried upon my poor cows to produce it. As to its calling out *fcrofulous complaints*, I never knew an inftance of it; and unlefs it be in cafe of puftules, I fhould think it cannot be expected. I never knew any cutaneous difeafe follow it; and during my practifing this new mode, I have never thought it neceffary to give a dofe of phyfic either after or before inoculation. I muft not conclude without noticing a circumftance which has frequently happened to me when I have been neceffitated to ufe *ftale matter*, and which, at the time time of my being leſs experienced, would moſt probably have led me into fatal errors. Upon introducing a bit of thread, which has been impregnated with vaccine lymph *only a few days*, inflammation has quickly followed, and, to a haſty obſerver, not very unlike the true inflammation; this runs on five, ſix, and ſeven days, then it ſuperficially ſuppurates, and for the moſt part ſcurfs over; but occaſionally it has continued a ſore ſome time longer, producing a degree of illneſs, &c. but never the genuine cow-pox. This ſhews, that the ſpecific powers of the vaccine fluid are ſoon loſt by putrefaction, and that the part is affected in the ſame manner it would be by any other putrid animal ſubſtance placed under the cuticle; and all thoſe inſtances enumerated by practitioners, as having been ſuſceptible of ſmall-pox after inoculation with the vaccine, are to be traced to this ſource of deception. I am,

GENTLEMEN,

Your obedient humble ſervant,

